# Water vapour sorption properties of a family of square lattice topology porous coordination networks†

**DOI:** 10.1039/d5ce00385g

**Published:** 2025-07-24

**Authors:** Samuel M. Shabangu, Alan C. Eaby, Lilia Croitor, Andrey A. Bezrukov, Michael J. Zaworotko

**Affiliations:** a Department of Chemical Sciences, Bernal Institute, University of Limerick Limerick V94 T9PX Republic of Ireland xtal@ul.ie

## Abstract

Porous coordination networks (PCNs) such as metal–organic frameworks are of topical interest thanks to their potential utility as sorbents for gas and vapour separations and/or storage. Interpenetrated PCNs, some of which offer promise for gas separations, remain relatively understudied in the context of water vapour sorption. Herein, we report an in-depth study of the water vapour sorption properties of a family of square lattice topology (**sql**) PCNs of general formula **sql-[bipy,squa]-M-aqua** (**sql-M-aqua**, M = Mn, Co, Ni, Zn, bipy = 4,4-bipyridine, squa = squarate). This family, several of which have been previously reported (Co, Ni, Mn), exist as rectangular grids that exhibit 2-fold inclined interpenetration, thereby forming ultramicroporous 3D supramolecular networks. Water vapour sorption studies of **sql-M-aqua** (M = Mn, Co, Ni, Zn) revealed S-shaped water vapour sorption isotherms with steps consistently below 10% relative humidity (RH) and little hysteresis. Such properties are pertinent to atmospheric water harvesting in arid regions (<30% RH). Water vapour humidity swing experiments (0–30% RH, 300 K) indicated hydrolytic stability for **sql-M-aqua** (M = Mn, Co, Ni, Zn) and retention of working capacity over 100 sorption/desorption cycles. **sql-M-aqua** (M = Mn, Co, Ni, Zn) also exhibit CO_2_/N_2_ selectivity.

## Abstract 2

The family of coordination networks **sql-M-aqua** (M = Mn, Ni, Co, Zn) each exhibit an S-shaped water sorption isotherm, fast uptake kinetics, and hydrolytic stability over 100 cycles, ideal for atmospheric water harvesting at 0–30% relative humidity.

## Graphical abstract

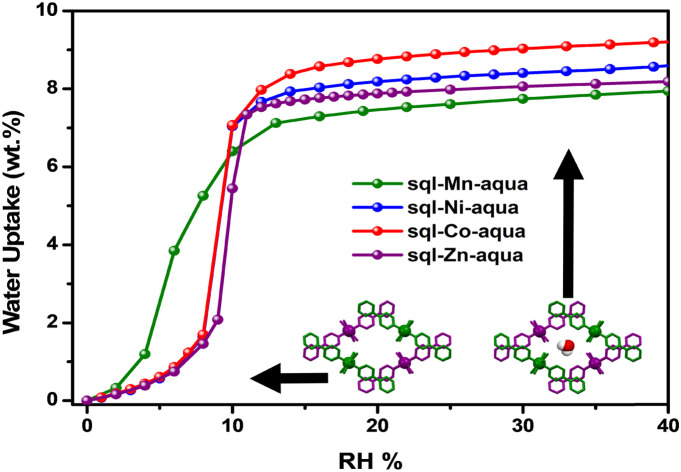

## Introduction

Crystal engineering^1–3^ of porous coordination polymers (PCPs)^4^ such as metal–organic frameworks (MOF)^5,6^ or porous coordination networks (PCNs)^7^ represents a topical area of research owing to their potential utility for global challenges such direct air capture of CO_2_,^8,9^ separation of CO_2_ from N_2_ (*e.g.* flue gas remediation) or CH_4_ (for natural gas refinement)^10,11^ and atmospheric water harvesting (AWH).^12,13^ PCNs are typically comprised of metal-based molecular building blocks (MBBs)^14^ connected by organic linker ligands. Their high degree of modularity enables systematic crystal engineering studies of structure/function relationships.^15^ In such a manner, sorption properties can be fine-tuned by modifying pore size and chemistry using the node-and-linker approach introduced by Hoskins and Robson.^2^ The high structural and compositional diversity enabled by this modularity has resulted in >125 000 PCNs currently archived in the Cambridge Structural Database (CSD).^16^

Selective guest inclusion by PCNs necessitates pores and/or cavities of appropriate size and compatible chemical functionality. Three generations of PCNs were outlined by Kitagawa:^17^ first-generation materials, which irreversibly collapse after guest removal and are therefore unsuitable for sorption applications; second-generation materials that are rigid, *i.e.* retain their porous structure or exhibit permanent porosity after activation and sorption/desorption cycles; third-generation sorbents which are flexible, *i.e.* display dynamic structural behaviour triggered by external stimuli such as exposure to gases or vapours.^18^ The design principles that govern porosity in PCNs are now quite well-defined^19^ and the most prevalent topologies, all of which are highly amenable to crystal engineering studies, are 2-dimensional square lattice (**sql**, 2D),^20^ 3-dimensional primitive cubic (**pcu**, 3D)^21^ and diamondoid (**dia**, 3D).^22^

PCNs with **sql** topology can be ideal platforms for the gaining insight into sorption properties as metal, linker ligand, anion and guest can all be substituted.^15,23–25^ Further, **sql** nets can be readily designed by self-assembly of 4-connected metal centres and ditopic linker ligands. Linker ligands can be classified by their coordinating moieties: N-donor only (*e.g.* 4,4-bipyridine, bipy); dicarboxylate donor only (*e.g.* 1,4-benzenedicarboxylate); mixed N-donor/carboxylate (*e.g.* isonicotinate).^26^ These types of linkers can also coexist in “mixed linker” PCNs. Our recent analysis of crystal structures deposited in the TOPOS TTO∩CSD^27,28^ databases revealed >9000 **sql** network structures archived in the CSD^26^ with >1300 being mixed linker networks.^26^ Mixed-linker PCNs offer a versatile platform, wherein judicious selection of electron-donating and electron-accepting linkers enables donor–acceptor architectures with enhanced charge-transfer characteristics to modulate the electronic structure of the framework.^29^ Furthermore, our group has reported on the **sql** net [Zn(Ria)(bphy)]^30^ and the effect of different carboxylate linkers upon water vapour sorption properties.

Interpenetration can occur in PCNs and **sql** nets and can be enabled by the use of longer linkers, significantly reducing porosity.^31,32^ An archetypal example of an interpenetrated PCN is the **sql** net [Zn(bipy)_2_(H_2_O)_2_]SiF_6_. The Cu variant of this **sql** net exists in [Cu(bpy)_2_(BF_4_)_2_], **ELM-11**,^33^ which was observed to exhibit guest-induced switching behaviour in the presence of gases such as CH_4_, CO_2_, C_2_H_2_, N_2_, O_2_ and *n*-butane. The mechanism of switching in this and related PCNs can be attributed to clay-like expansion/shrinkage between adjacent layers of **sql** planes enabled by layer–guest interactions.

**Fig. 1:**
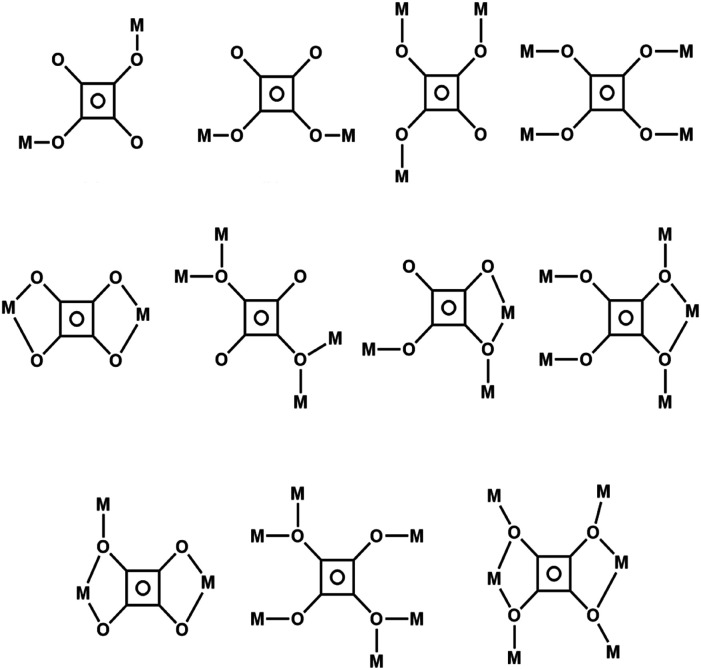
Coordination modes of squa ligands in the construction of CNs structures.

Carboxylate linker ligands include deprotonated squaric acid (3,4-dihydroxycyclobut-3-ene-1,2-dione, H_2_squa), C_4_O_4_^2−^, which is promiscuous in terms of the motifs it exhibits (Fig. 1).^34^ Our analysis of the CSD (2D MOF subset) revealed that 42 **sql** topology CNs based on squa linkers have been reported, 6 of which are nonporous single linker **sql** nets (type 1-a),^35^ whereas 9 examples are porous mixed-linker **sql** nets (type II-ab)^35^ (Table S1†). Our interest in these type II-ab **sql** nets was prompted by their ultramicroporous nature, resulting from interpenetration, and the presence of channel water molecules in crystal structures, suggesting water stability. Water vapour sorption studies of **sql** nets are to our knowledge limited to six examples: {[Cu(bpy)_2_(5-H_2_sip)_2_]·(H_2_O)_6_}_*n*_,^36^ CID-5/CID-6,^37^ MCID-1,^38^ [Zn(Ria)(bphy)],^30^ sql-(1,3-bib)(ndc)-Ni (ref. 39) and sql-(azpy)(pdia)-Ni,^40^ all of which are non-interpenetrated. We herein report our investigation of the gas and water vapour sorption properties of the family of type II-ab **sql** CNs sql-[bipy,squa]-M-aqua, **sql-M-aqua** (M = Co, Ni, Mn, Zn). These CNs are ultramicroporous^41^ and exhibit 2-fold interpenetration, previous reports having addressed structural (M = Co, Ni, Mn)^42,43^ and selected sorption properties (CO_2_, 195 K; N_2_, 77 K, water vapour) for M = Cd (Table S1†),^44^ An in-depth analysis on the water vapour sorption properties of the **sql-M-aqua** family to address key performance parameters such as water sorption kinetics and recyclability is reported herein. In addition, we report a new member (M = Zn) to further study the effect of metal substitution on properties given the different water sorption properties in the family **M**_**2**_**Cl**_**2**_**(BTDD)** (M = Mn, Co, and Ni).^45^

## Results and discussion

**Fig. 2:**
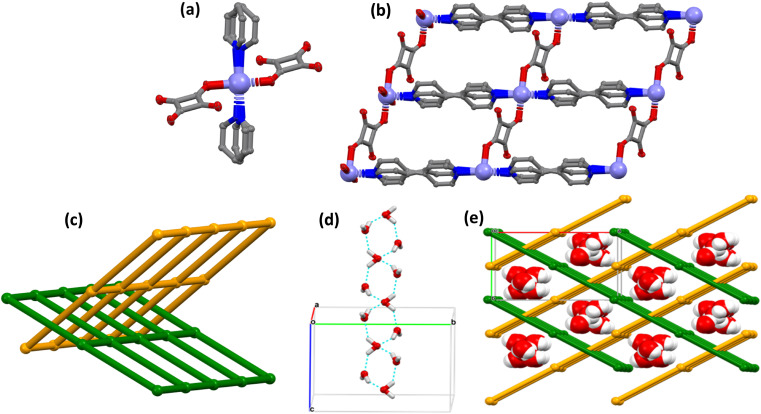
(a) The 4-connected octahedral geometry molecular building block (MBB) in **sql-M-aqua**, (b) 2D rectangular grid net, (c) 2-fold interpenetrated **sql** nets, and displacement ellipsoids in are drawn at the 50% probability level, (d) 1D hydrogen bonded water chain (M = Zn) and (e) 3D supramolecular network of **sql-M-aqua** (M = Mn, Co, Ni, Zn). Bipy, squa, some aqua ligands and hydrogen atoms have been omitted for clarity.

**sql-M-aqua** (M = Mn, Co, Ni, Zn), were prepared following a modified hydrothermal procedure (see ESI†).^42,43^ Attempts to synthesise the Fe and Cu analogues were unsuccessful. Single crystals of **sql-Zn-aqua** crystallised in the monoclinic space group *C*2/*c* (Table S2†) but suitably sized single crystals of **sql-M-aqua** (M = Mn, Co, Ni) could not be prepared under the same conditions. The 4-connected octahedral MBB in as-synthesised **sql-Zn-aqua** comprises two nitrogen donors from bipy ligands, two oxygen donors from two bridging squarate ligands and two coordinated aqua ligands (Fig. 2a).^43^ As illustrated in Fig. 2b, bipy and squarate anions serve as linkers that bridge metal cations to form the expected rectangular **sql** grids (Fig. 2b). Stacking of the resulting 2D sheets results in two sets of layers oriented in the [1, 1, 0] and [−1, 1, 0] directions Fig. 2c. Lattice water molecules lie in the 1D channel along the *c* axis, forming hydrogen-bonded chains with a rhombohedral motif (Fig. 2d) that in effect cross-link 2D **sql**-layers into a 3D supramolecular network, Fig. 2e, through O–H⋯O hydrogen bonds with coordinated O atoms of squarate anions (Table S3†). Crystal packing is further defined by CH⋯π interactions involving the pyridine rings of adjacent bipy layers. **sql-M-aqua** (M = Mn, Co, Ni, Zn) can therefore be considered a variant of the prototypal interpenetrated **sql** net, **[Zn(bpy)**_**2**_**(H**_**2**_**O)**_**2**_**]**_***n***_**·SiF**_**6**_, however, **sql-M-aqua** (M = Mn, Co, Ni, Zn) is charge neutral and porous, in contrast to **[Zn(bpy)**_**2**_**(H**_**2**_**O)**_**2**_**]**_***n***_**·SiF**_**6**_, in which the pores are filled by extra-framework anions. PXRD diffractograms of **sql-M-aqua** (M = Mn, Co, Ni, Zn) match those calculated from their crystal structures (Fig. S1†).^21,43^ Thermogravimetric analysis (TGA, Fig. S2†) revealed mass losses of 10% (onset temperature *T*_on_ = 337 K), 9% (*T*_on_ = 340 K), 8% (*T*_on_ = 341 K) and 9% (*T*_on_ = 347 K) for **sql-Mn-aqua**, **sql-Co-aqua**, **sql-Ni-aqua** and **sql-Zn-aqua**, respectively. These mass losses correspond to removal of 3 molecules of H_2_O per formula unit (H_2_O/FU). The second step corresponds to removal of aqua ligands with *T*_on_ = 454 K, 468 K, 463 K and 461 K **for sql-Mn-aqua**, **sql-Co-aqua**, **sql-Ni-aqua** and **sql-Zn-aqua**, respectively. *T*_on_ for the second step increased in the following order: Mn < Zn < Ni < Co. This indicates relative bond strength between aqua ligands and the metal ions and is consistent with what was previously observed for **sql-M-aqua** (Co,Ni).^43^ These assertions are supported by variable temperature powder X-Ray diffraction (VT-PXRD) under N_2_ gas flow (Fig. S3–S6†). VT-PXRD diffractograms reveal that PXRD peaks remain largely unchanged during heating from 298 K to 333 K, indicating retention of structure during desorption of lattice water.^43^ Further heating to 523 K (**sql-Mn-aqua**, **sql-Zn-aqua**, **sql-Ni-aqua**, **sql-Co-aqua**) resulted in structural transformations. This further heating also led to a decline in crystallinity of **sql-Mn-aqua**, **sql-Co-aqua** and **sql-Zn-aqua** as evidenced by broadened PXRD peaks. The heat of dehydration associated with lattice water and removal of aqua ligands was measured using differential scanning calorimetry (DSC, Fig. S7†) and two exothermic events were observed: *T*_on_ = RT (−39 kJ mol^−1^); *T*_on_ = 368 K (−47 kJ mol^−1^), respectively. Notably, after exposure to 95.5% relative humidity (RH) for 1 h, the starting material was regenerated and exhibited thermal events with comparable dehydration energies: *T*_on_ = RT (−41 kJ mol^−1^); *T*_on_ = 371 K (−40 kJ mol^−1^). These observations support reversibility of the hydration–dehydration process. The ease with which lattice water was removed from the powdered samples prompted us to revisit our SCXRD study of **sql-Zn-aqua**. The crystal structure determined from heating **sql-Zn-aqua** to 373 K under a dry N_2_ stream, followed by cooling to 298 K, revealed that lattice water molecules had indeed been desorbed without significant changes to the crystal structure (Table S4†). Several attempts to obtain a crystal structure of the high-temperature phases were made by heating single crystals *in situ*, but they resulted in polycrystalline samples.

### Gas sorption studies

To evaluate gas sorption properties, powdered samples of **sql-M-aqua** (M = Mn, Co, Ni, Zn) were activated at 308 K under dynamic vacuum for 2 h, conditions that did not result in removal of aqua ligands. We conducted CO_2_ (195 K) and N_2_ (77 K) gas sorption measurements upon **sql-M-aqua** (M = Mn, Co, Ni, Zn). Type I^46^ CO_2_ adsorption isotherm profiles with modest uptakes of 35 cm^3^ g^−1^, 45 cm^3^ g^−1^, 43 cm^3^ g^−1^ and 41 cm^3^ g^−1^ for **sql-Mn-aqua**, **sql-Co-aqua**, **sql-Ni-aqua** and **sql-Zn-aqua**, respectively (Fig. S8†), with minimal N_2_ adsorption (Fig. S9†). The recorded CO_2_ uptakes at 760 mmHg and 273 or 298 K (Fig. S10†) were as follows: **sql-Mn-aqua** (19 cm^3^ g^−1^, 16 cm^3^ g^−1^), **sql-Co-aqua** (19 cm^3^ g^−1^, 17 cm^3^ g^−1^), **sql-Ni-aqua** (20 cm^3^ g^−1^, 15 cm^3^ g^−1^) and **sql-Zn-aqua** (20 cm^3^ g^−1^, 18 cm^3^ g^−1^) respectively. Negligible N_2_ uptake at 298 K was observed (Fig. S10†), suggesting the possibility of separating CO_2_ from N_2_. We attribute the negligible uptakes in the N_2_ isotherms to surface adsorption driven by sieving of N_2_ (the kinetic diameter of N_2_, 3.6 Å, is larger than that of CO_2_, 3.3 Å). The N_2_ uptakes are therefore subject to anomalies caused by different particle size distributions and/or incomplete equilibration. Ideal absorbed solution theory (IAST)^47^ calculations can serve as an indicator for separation performance. In this context, CO_2_/N_2_ (15 : 85) selectivity was calculated using Ideal IAST as determined from 273 K and 298 K data (Fig. S11†). These values were determined to be 4.6, 18.2, 42.1 and 54.8 for **sql-Mn-aqua**, **sql-Co-aqua**, **sql-Ni-aqua** and **sql-Zn-aqua**, respectively. The selectivity observed is lower than then most selective CO_2_/N_2_ sorbents under similar conditions as exemplified by **SIFSIX-3-Zn** (1538),^48^**SIFSIX-3-Ni** (1438),^49^**NbOFFIVE-1-Ni** (6528),^50^**SIFSIX-2-Cu-i** (71.9)^48^**Zeolite 13X** (562)^51^**mmen-Mg**_**2**_**(dobpdc)** (200),^52^**MOF-74-Mg** (61.1).^53^ The observed CO_2_ selectivity can be attributed to **sql-M-aqua** (M = Mn, Co, Ni, Zn) being ultramicroporous but with weaker sorbent-sorbate interactions when compared to benchmark CO_2_ sorbents such as hybrid ultramicroporous materials.^40^

### Water vapour sorption studies

**Fig. 3:**
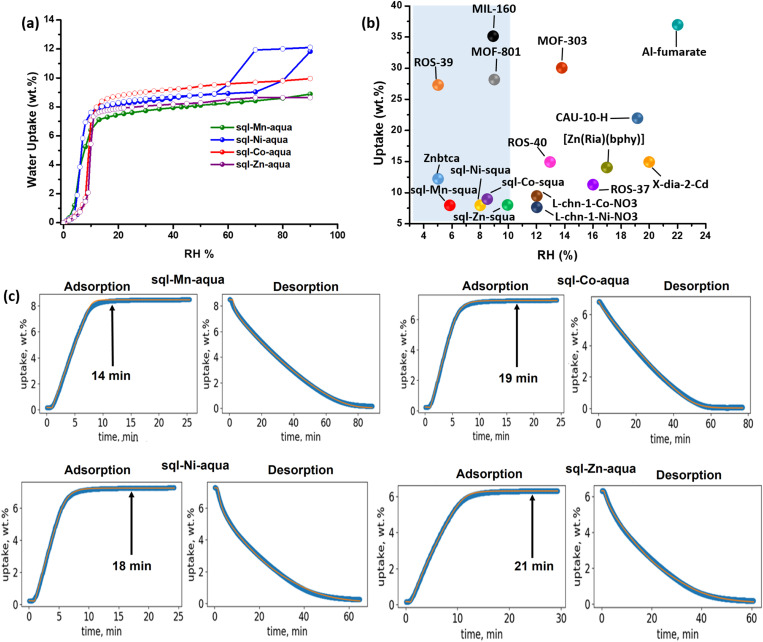
(a) Water vapour sorption isotherm collected (300 K) by dynamic vapour sorption (DVS), (b) comparison of inflection points between reported AWH sorbents (the area shaded in blue represents the preferred adsorption step for an AWH sorbent to ensure fast adsorption) and (c) 0–30% RH humidity swing kinetics data for **sql-M-aqua** (M = Mn, Co, Ni, Zn).

Dynamic water vapour sorption (DVS) experiments were conducted at 300 K after activating samples of **sql-M-aqua** (M = Mn, Co, Ni, Zn) at 353 K for 2 h under dry air. The sorption isotherms revealed steps at the following inflection points: 6% RH for **sql-Mn-aqua**; 8% RH for **sql-Co-aqua**; 8% RH for **sql-Ni-aqua**; 10% RH for **sql-Zn-aqua**. Water uptakes of *ca.* 8.5 wt% (3H_2_O/, FU), *ca.* 9 wt% (3H_2_O/FU), 8.6 wt% (3H_2_O/FU) and 8.2 wt% (3H_2_O/FU), were observed for **sql-Mn-aqua**, **sql-Co-aqua**, **sql-Ni-aqua** and **sql-Zn-aqua**, respectively. The desorption profiles revealed negligible hysteresis (Fig. 3a) except for **sql-Ni-aqua**, which displayed hysteresis resulting from evaporation of this interparticle water at *ca.* 55% RH. Variable-temperature PXRD data (Fig. S5†) indicate that no phase change occurred during this step, the network structure being unchanged upon heating of as-synthesised crystals. The only phase transformation observed corresponds to loss of aqua ligands in **sql-M-aqua** (M = Mn, Co, Ni, Zn), as evidenced by the appearance of new PXRD peaks above 373 K. Interestingly, the observed stepped isotherms within the range 0–30% RH are relevant to AWH.^12,54–56^ Such S-shaped isotherm profiles are consistent with either a pore-filling (type V^57^) or a structural transformation (phase switching^58^) mechanism. Since **sql-M-aqua** (M = Co, Ni, Zn) maintained their structures following removal of channel water, as evidenced by TGA analysis and VT-PXRD (Fig. S2–S6†), we can assert that sorption occurred by pore-filling.^57^ Initial uptake observed at RH below the step in the sorption isotherm corresponds to hydrogen-bond interactions between lattice water molecules and the uncoordinated oxygen atoms of squa anions. Further loading resulted in the formation of an infinite network of hydrogen-bonded lattice water molecules with O_water_–O_water_ distances ranging from 2.7–2.9 Å (Fig. S12†). The relative strength of the water–water interactions can be inferred from the enthalpies of adsorption at 50% uptake, Δ*H*_ads_, which are representative of the enthalpies of the pore-filling mechanism (see Fig. S13–S17†): −63 ± 3 kJ mol^−1^, −58 ± 6 kJ mol^−1^, −56 ± 6 kJ mol^−1^, −57 ± 6 kJ mol^−1^ for **sql-M-aqua** (M = Mn, Co, Ni, Zn) (Fig. S17†). These values compare with other MOFs that have Δ*H*_ads_ values ranging from 36–75 kJ mol^−1^.^59^ To evaluate recyclability, humidity swing cycling tests (0–30–0% RH, 300 K) were conducted on 11 mg samples. **sql-M-aqua** exhibited hydrolytic stability over 100 cycles, retaining both water sorption capacity and crystallinity as evidenced by PXRD patterns collected before and after cycling experiments, none of which showed significant changes in peak positions or intensities (Fig. S18 and S19†). With respect to assessing performance for atmospheric water harvesting, adsorption/desorption rates are key performance parameters.^54,60–62^ However, water vapour sorption kinetics are rarely reported.^58,63–67^ Our group has developed an isotherm-based kinetics model^60^ that correlates water vapour sorption isotherms with sorption kinetics. This model helps to explain differences in sorption kinetics for sorbents including both rigid, *e.g.***ROS-037**,^68^**ROS-039**,^69^**ROS-040**,^70^**MOF-303**,^62^**MIL-160**,^71^**CAU-10-H**,^72^**Al-fumarate**^73^ and flexible, *e.g.***X-dia-2-Cd**,^58^**Znbtca**^74^ and **sql-(azpy)(pdia)-Ni** sorbents. That **sql-M-aqua** exhibited inflection points at <10% RH means that the model^60^ predicted fast adsorption rates, similar to the adsorption kinetics of **ROS-039** (ref. 69) and **MIL-160**.^71^ Very few AWH sorbents exhibit steps at such low RH (Fig. 3b). Humidity-swing experiments conducted on **sql-M-aqua** under AWH-relevant conditions (0 to 30% RH at 300 K) revealed fast adsorption relative to desorption: **sql-Mn-aqua** (14 min adsorption, 78 min desorption); **sql-Co-aqua** (19 min adsorption, 65 min desorption); **sql-Ni-aqua** (18 min adsorption, 63 min desorption); **sql-Zn-aqua** (21 min adsorption, 58 min desorption), Fig. 3c. The relatively fast adsorption rates for **sql-M-aqua** are consistent with our isotherm-based kinetic model, which shows that adsorption kinetics are dependent on the position of the inflection point in the water vapour isotherm.^60^ Whereas metal substitution in **sql-M-aqua** (M = Mn, Co, Ni, Zn) had little impact upon the S-shaped isotherm, the same cannot be said for **sql-Cd-aqua**, which was reported to exhibit a moderately hydrophilic Type I isotherm unsuitable for AWH.^44^ The subtle, but notable differences in water vapour sorption properties in Fig. 3a prompted us to revisit the crystal structures of the water loaded phases in **sql-M-aqua** (M = Mn, Co, Ni, Zn, Cd). Changing the metal node led to differences in the channel dimensions (Fig. S12†) and thus the structures of the available voids. Furthermore, although the water–water hydrogen bond distances are comparable, *ca.* 2.8 A, the water network structures exhibit subtle differences in host–guest and guest–guest interactions (Table S3 and Fig. S12†). Specifically, the positioning of the water chains in the porous structures varies between the different analogues (Fig. S12†). Such differences are known to lead to variations in water vapour sorption properties^75^ and could explain the slight differences in the position of the inflection point in the sorption isotherms.

## Conclusions

In summary, the work presented herein underscores the role of crystal engineering in the development of PCNs for gas and vapour sorption applications. By synthesising a family of **sql** PCNs **sql-M-aqua** (M = Mn, Co, Ni, Zn), we reveal the effect of metal node substitution on the sorption properties of these materials. The observed S-shaped water vapour isotherms, coupled with minimal hysteresis and hydrolytic stability, suggest potential utility for AWH at low-humidity (10–30% RH). Additionally, retention of working capacity over 100 sorption–regeneration cycles highlights the robustness of these materials. Overall, these findings not only advance our understanding of structure–function relationships in desiccants but also reinforce the value of systematic crystal engineering studies. Whereas **sql** PCNs are abundant in the CSD, only a handful meet the performance criteria for AWH. **sql-M-aqua** (M = Mn, Co, Ni, Zn) are now included in this select group of sorbents.

## Author contributions

The manuscript was written through contributions of all authors. All authors have given approval to the final version of the manuscript. CRediT: S. M. S., A. A. B., A. C. E.: conceptualization, investigation, methodology, writing-original draft, review and editing; L. C.: investigation, writing-review and editing; M. J. Z.: funding acquisition, formal analysis, writing-review and editing.

## Conflicts of interest

There are no conflicts of interest to declare.

## Supplementary Material

CE-027-D5CE00385G-s001

CE-027-D5CE00385G-s002

## Data Availability

The data supporting the findings of this study are available in the ESI† or from the authors upon request.
