# Supplementary material for: Water vapour sorption properties of a family of square lattice topology porous coordination networks
Source: CrystEngComm. 2025 Jul 24;27(34):5669–75. doi: 10.1039/d5ce00385g (PMC12319667; doi:10.1039/d5ce00385g)
Supplement: CE-027-D5CE00385G-s001 [file CE-027-D5CE00385G-s001.pdf]

Supplementary Information (SI) for CrystEngComm.
This journal is © The Royal Society of Chemistry 2025

Electronic Supplementary Information (ESI)

Water vapour sorption properties of a family of square lattice topology porous coordination networks

Samuel M. Shabangu, Alan C. Eaby, Lilia Croitor, Andrey A. Bezrukov and Michael J. Zaworotko*[a]

[a] Department of Chemical Sciences, Bernal Institute, University of Limerick, Limerick V94 T9PX, Republic of Ireland

Table of Contents

Table of contents.. 2

Methods.. 4

S1. Materials and synthesis.. 4

S2. Single crystal X-ray Diffraction 4

S3. Powder X-ray Diffraction Measurements.. 4

S4. Thermogravimetric Analysis.. 4

S5. Variable temperature Powder diffraction Measurements.. 5

S6. Cryogenic Gas sorption Measurements.. 5

S7. Dynamic vapour sorption.. 5

S8. CSD Database search 6

References.. 22

List of Tables

Table S1: CSD survey on porous **sql** CNs sustained by squa and N-donor ligands (mixed linker, Type II-ab).............. 6

Table S2: Selected crystallographic details for **sql-M-aqua (M = Mn, Co, Ni, Zn)**.. 7

Table S3: Comparison of Hydrogen bond distances (Å) between clathrated water molecules and sql-grid.............. 8

Table S4: Selected crystallographic details for **sql-Zn-aqua-activated**.. 12

List of Figures

Fig.S1**:** PXRD diffractograms of **sql-M-aqua** (M=Mn,Co,Ni,Zn) 8

Fig.S2: TGA curves of **sql-M-aqua** (M=Mn,Co,Ni,Zn) 8

Fig.S3-S6. VT-PXRD diffractograms of **sql-M-aqua** (M=Mn,Co,Ni,Zn) 9/10

Fig.S7**:** DSC thermograms of the heating segments for **sql-Zn-aqua** 11

Fig.S8: 195 K CO_2 adsorption and desorption isotherms measured for **sql-M-aqua** (M=Mn,Co,Ni,Zn) 12

Fig.S9: N_2 isotherms at 77K for **sql-M-aqua** (M=Mn,Co,Ni,Zn) 13

Fig.S10. CO_2 and N_2 isotherms at 273 and 298 K for **sql-M-aqua** (M=Mn,Co,Ni,Zn) 13

Fig.S11**.** IAST selectivities of **sql-M-aqua** (M=Mn,Co,Ni,Zn) at 298 K for CO_2/N_2 and compositions of 15:85, plotted as a function of pressure.. 14

Fig.S12: Comparison of guest-guest interactions in **sql-M-aqua** (Mn,Ni,Co,Zn,Cd).. 15

Fig.S13**:** Water vapour sorption isotherms for **sql-M-aqua** (M= Mn, Co, Ni, Zn) measured at 10 °C, 27 °C and 40 °C.. 16

Fig.S14: Water vapour adsorption isotherms used for calculating Δ*h* recorded at 10 °C (top), 27 °C (middle,) and 40 °C (bottom, red squares).. 17

Fig. S15: Water vapour adsorption isotherms used for calculating Δ*h* recorded at 10 °C (top), 27 °C (middle,) and 40 °C (bottom, red squares).. 18

Fig.S16: Adsorption isosteres used to calculate enthalpy of adsorption (Δ*h*) for **sql-M-aqua** (M= Mn, Co, Ni, Zn)........ 19

Fig.S17: Enthalpy of adsorption (Δ*h*) with respect to water uptake for **sql-M-aqua** (M= Mn, Co, Ni, Zn)...................... 20

Fig.S18: 100 regeneration cycles on 11 mg sample for **sql-M-aqua** (M= Mn, Co, Ni, Zn)... 21

Fig.S19: PXRD of **sql-M-aqua** (M=Mn,Co,Ni,Zn) after cycling experiment... 21

S1. Materials and synthesis

The reagents and solvents were commercially available and were used without further purification.

Synthesis of **sql-Mn-aqua**: $Mn(NO_3)_2 \cdot 4H_2O$ (0.0251 g, 0.1 mmol), $H_2C_4O_4$ (0.0114 g, 0.1 mmol), bipy (0.0171 g, 0.1 mmol) and deionized water (6 mL) was allowed to proceed at 100 °C for 12 h in a teflon reactor.

Synthesis of **sql-Co-aqua**: $Co(NO_3)_2 \cdot 6H_2O$ (0.0292 g, 0.1 mmol), $H_2C_4O_4$ (0.0114 g, 0.1 mmol), bipy (0.0171 g, 0.1 mmol) and deionized water (6 mL) was allowed to proceed at 100 °C for 12 h in a Teflon reactor.

Synthesis of **sql-Ni-aqua**: $Ni(NO_3)_2 \cdot 6H_2O$ (0.0291 g, 0.1 mmol), $H_2C_4O_4$ (0.0114 g, 0.1 mmol), bipy (0.0171 g, 0.1 mmol) and deionized water 6 mL was allowed to proceed at 100 °C for 12 h in a Teflon reactor.

Synthesis of **sql-Zn-aqua**: $Zn(NO_3)_2 \cdot 6H_2O$ (0.0297 g, 0.1 mmol), $H_2C_4O_4$ (0.0114 g, 0.1 mmol), bipy (0.0171 g, 0.1 mmol) and deionized water (6 mL) was allowed to proceed at 100 °C for 12 h in a Teflon reactor.

S2. Single crystal X-ray Diffraction Measurements

High quality single crystals of **sql-Zn-aqua** were chosen for single crystal X-ray diffraction measurements. Diffraction data for **sql-Zn-aqua** (150 K) was collected on a Bruker Quest diffractometer equipped with a IµS microfocus X-ray source (Cu Kα, λ = 1.54178 Å; Mo Kα, (λ = 0.71073 Å) and CMOS detector. In all cases, data was indexed, u8)=p[‘4ntegrated and scaled using Bruker SAINT software.[1] Space group determination was performed simultaneously with structure solution using SHELXT[3] intrinsic phasing methods through the X-Seed[4,5] graphical user interface. Zinc, carbon, nitrogen, oxygen and hydroxo hydrogen atoms of the host were refined anisotropically using SHELXL, using full-matrix least squares minimization.[2] Host hydrogen atomic positions were calculated using riding models. Selected crystallographic parameters are reported in Table S2 and S4. Pore volumes and geometries (pore limiting diameters and maximum pore diameters) were calculated using the Pore Analyzer (default settings) feature in Mercury.[3] Crystal structures and void volumes were visualized using Mercury.[3] Difference electron density maps were calculated using OLEX2.[4]

S3. Powder X-ray Diffraction Measurements

Diffractograms were recorded using a PANalytical Empyrean™ diffractometer equipped with a PIXcel3D detector operating in scanning line detector mode with an active length of 4 utilizing 255 channels. The diffractometer is outfitted with an Empyrean Cu LFF (long fine-focus) HR (9430 033 7310x) tube operated at 40 kV and 40 mA and CuKα radiation (λα = 1.540598 Å) was used for diffraction experiments. Continuous scanning mode with the goniometer in the theta-theta orientation was used to collect the data. Incident beam optics included the Fixed Divergences slit with anti-scatter slit PreFIX module, with a 1/8° divergence slit and a 1/4° anti-scatter slit, as well as a 10 mm fixed incident beam mask and a Soller slit (0.04 rad). Divergent beam optics included a P7.5 anti-scatter slit, a Soller slit (0.04 rad) and a Ni-β filter. In a typical experiment, 25 mg of sample was dried, ground into a fine powder and was loaded on a zero background silicon disks. The data was collected from 5°–40° (2θ) with a step-size of 0.0131303° and a scan time of 30 seconds per step. Crude data were analyzed using the X’Pert HighScore Plus™ software V 4.1 (PANalytical, The Netherlands).

S4. Thermogravimetric Analysis

Thermogravimetric analyses (TGA) were performed under N_2 using a TA Instruments Q50 system. Samples were loaded into aluminium sample pans and heated at a rate of 10 K min^{-1} from room temperature to 500 °C.

S5. Variable temperature Powder diffraction Measurements

Diffractograms at different temperature were recorded using a PANalytical X'Pert Pro-MPD diffractometer equipped with a PIXcel3D detector operating in scanning line detector mode with an active length of 4 utilizing 255 channels. Anton Paar TTK 450 stage coupled with the Anton Paar TCU 110 Temperature Control Unit was used to record the variable temperature diffractograms. The diffractometer is outfitted with an Empyrean Cu LFF (long fine-focus) HR (9430 033 7300x) tube operated at 40 kV and 40 mA and CuKα radiation (λα = 1.54056 Å) was used for diffraction experiments. Continuous scanning mode with the goniometer in the theta-theta orientation was used to collect the data. Incident beam optics included the Fixed Divergences slit, with a 1/4° divergence slit and a Soller slit (0.04 rad). Divergent beam optics included a P7.5.

S6. Gas sorption Measurements

Before performing the gas sorption experiments, a freshly prepared sample of **sql-Mn-aqua, sql-Co-aqua**, **sql-Ni-aqua** and **sql-Zn-aqua** were placed in a quartz tube and degassed under high vacuum using a Smart VacPrep instrument at 308 K for 2 h to remove any remaining solvent molecules. Isotherms were measured using a Micromeritics 3Flex sorption analyser. Gases were used as obtained from BOC Gases (Ireland), with the following certified purities: research-grade CO_2 (99.995%) N_2 (99.9995%%). Bath temperature of 195 K were maintained using dry ice–acetone slurry. The temperature at 77 K was maintained using a 4 L Dewar filled with liquid nitrogen, CO_2 (99.995%), N_2 (99.9995%%). Bath temperatures of 273 and 298 K were precisely controlled with a Julabo ME (v.2) recirculating control system containing a mixture of ethylene glycol and water.

S7. Dynamic vapour sorption

Water vapor sorption isotherm measurements were performed using Adventure dynamic vapor sorption (DVS) instrument manufactured by Surface Measurement Systems. The instrument gravimetrically measures water vapor uptake using air as a carrier gas. Digital mass flow controllers regulate flows of dry and saturated gases. Relative humidity is generated by precisely mixing dry and saturated gas flows in desired flow ratios which produce expected relative humidity. Pure water was used to generate water vapor for these measurements and temperature was maintained at 300 K by enclosing the system in a temperature-controlled incubator. The mass of the sample was determined by a high-resolution microbalance with a precision of 0.01 µg. Microbalance has symmetric configuration with two branches of the balance being exposed to the same gas and being kept at the same temperature, which allows negation of buoyancy and drag effects. Instrument allows measurement of 2 samples in parallel. Isotherm measurements were performed on approximately 11 mg of sample powder. 400 sccm min^{-1} total flow was used for the measurements at 300 K. For each isotherm point, dm/dt < 0.01 %/min was used as criteria of reaching equilibrium.

Water vapor sorption cycling was performed at 300 K on a Surface Measurement Systems DVS adventure instrument using air as a carrier gas to gravimetrically measure the uptake and loss of vapor. The mass of the sample was determined by comparison to an empty reference pan and recorded by a high-resolution microbalance with a precision of 0.1 µg. Prior to the measurement, the sample (11 mg) was activated in dry air at 298 K for 120 minutes. Humidity swing was measured between two points 0 and 30 % RH. 100 cycles were subsequently performed.

Experimental sorption kinetics was measured on DVS adventure instrument using humidity swing experiment performed between two points for 0-30 on a 11 mg sample. Experimental sorption kinetics was modelled using isotherm-based kinetics model recently published by us.[5] Adsorption and desorption kinetics was modelled using Eqn. 2. RH_{bed} was determined from adsorption branch of the isotherm at the corresponding uptake. Two parameters were fitted: k and t0, where t0 was fitted in 0-3 minutes range, fitting parameters could be found in Figure 3.

$\frac{dw}{dt} = k \cdot (RH_{flow} - RH_{bed})$ Eqn.2

where w is uptake (wt.%), k is kinetics coefficient, RH_{flow} is relative humidity in the flow and RH_{bed} is relative humidity in sample bed.

S8. CSD Database search

A survey using CSD (v5.44)[6] search was conducted(2D MOF subset) on **sql** coordination networks (2D) sustained by squaric acid (dianion of 3,4-dihydroxycyclobut-3-ene-1,2-dione). The list of refcodes obtained was analysed using mercury[3] to assess mixed linker nets. Sorption results were also surveyed through published articles based on the refcode deposited.

Table S1: CSD survey on porous **sql** CNs sustained by squa and N-donor ligands (mixed linker, Type II-ab)

REFCODE	Metal	Sorption	N donor ligand	Reference
WUPZEM*	Mn	No	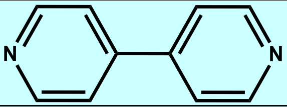	7
ULUKAN*	Co	No	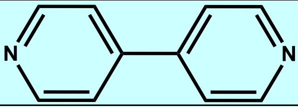	8
ULUKIV*	Ni	No	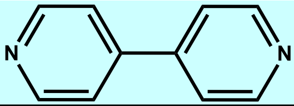	8
BARXIC	Fe	No	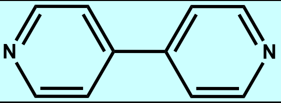	8
BICHEB	Cd	CO_2,N_2,H_2O	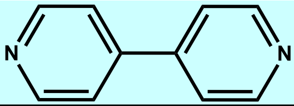	9
	Zn		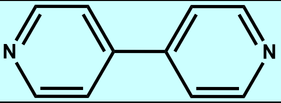	This work
ACIQEJ	Zn	No	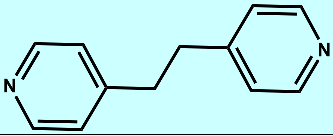	10
RAXNEK	Fe	No	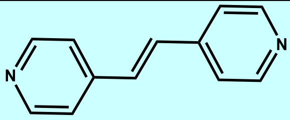	11
LEDJIP		No	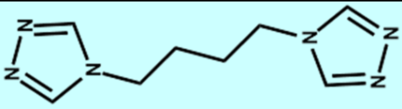	12
XUZXIB	Co	No	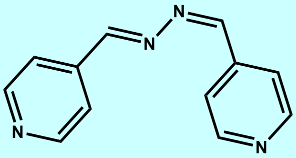	13

***evaluated in this work**

Table S2: Selected crystallographic details for **sql-M-aqua (M = Mn, Co, Ni, Zn)**

Unit Cell Parameters	sql-Co-aqua (REFCODE:ULUKAN)	sql-Ni-aqua (REFCODE:ULUKIV)	sql-Mn-aqua (REFCODE:WUPZEM)	sql-Zn-aqua (CCDC# 2390773)
Formula	$C_{14}H_{18}N_2O_9Co$	$C_{14}H_{18}N_2O_9Ni$	$C_{14}H_{18}N_2O_9Mn$	$C_{14}H_{18}N_2O_9Zn$
Formula weight (g/mol)	417.23	394.22	413.24	423.71
Temperature (K)	170 K	170 K	130 K	150K
Crystal system	Monoclinic	Monoclinic	Monoclinic	Monoclinic
Space group	$P2_1/c$	$P2_1/c$	$P2_1/c$	$C2/c$
a (Å)	18.937 (1)	18.271 (1)	18.716 (1)	19.9774(9)
b (Å)	11.342 (1)	11.340 (1)	11.544 (1)	11.3313(6)
c (Å)	8.0545 (5)	7.8946 (4)	8.1738 (5)	8.0463(3)
β (°)	91.83 (1)	90.69 (1)	90.536 (7)	109.438(2)
V (Å^3)	1725.3	1633.1	1766.0	1717.62
Z	4	4	4	4
GooF	1.094	1.106	1.027	1.005
R_1 [$I > 2\sigma(I)$]	0.0444	0.0319	0.0342	0.0545
WR_2 [all data]	0.1273	0.0858	0.0919	0.0884

Table S3. Comparison of Hydrogen bond distances (Å) between clathrated water molecules and sql-grid

	**sql-Mn-aqua**	**sql-Co-aqua**	**sql-Ni-aqua**	**sql-Zn-aqua**
O_{UW}-H...O_{CW}	-	-	2.890 (O9···O6)	-
O_{UW}-H...O_{US}	-	-	3.034 (O8···O2)	-
O_{UW}-H...O_{CS}	2.851 (O7···O1)<br>2.841 (O9···O4)	2.862 (O7···O1)<br>2.843 (O9···O4)	3.093 (O7···O1)	2.845 (O4···O1)

*UW-uncoordinated water molecule; CW-coordinated water molecule; US-uncoordinated squarate oxygen atom; CS-coordinated squarate oxygen atom

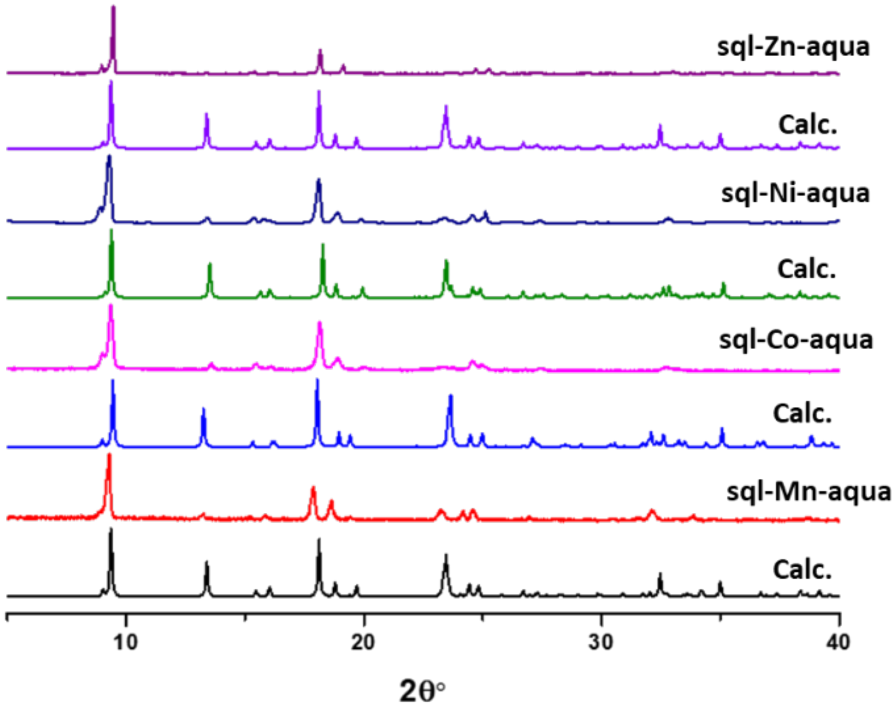

Figure S1: PXRD diffractograms of **sql-M-aqua** (M = Mn, Co, Ni, Zn).

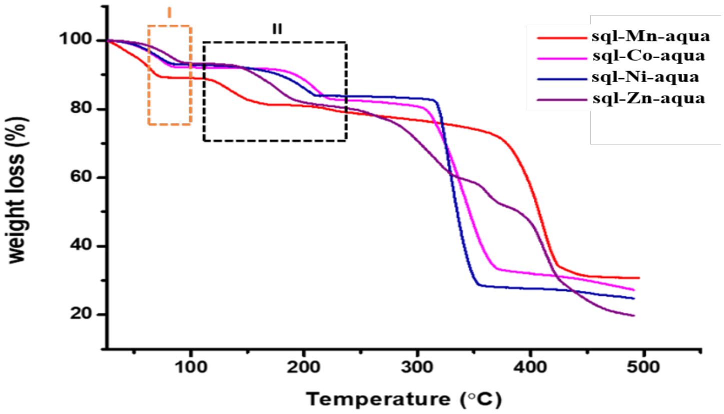

Figure S2: TGA curves of **sql-Mn-aqua**(red), **sql-Co-aqua** (pink), **sql-Ni-aqua** (blue) and and **sql-Zn-aqua** (purple) under N_2 flow and the corresponding H_2O lost during heating: (I) first step- 3 H_2O (channel water); (II) second step- 2 H_2O (aqua ligand).

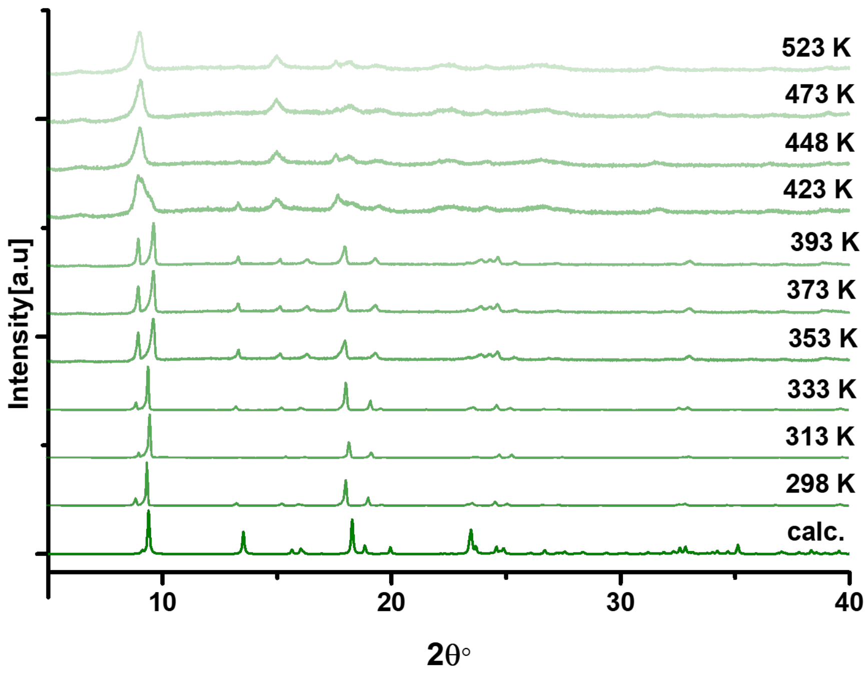

Figure S3: VT-PXRD diffractograms of **sql-Mn-aqua** under N_2.

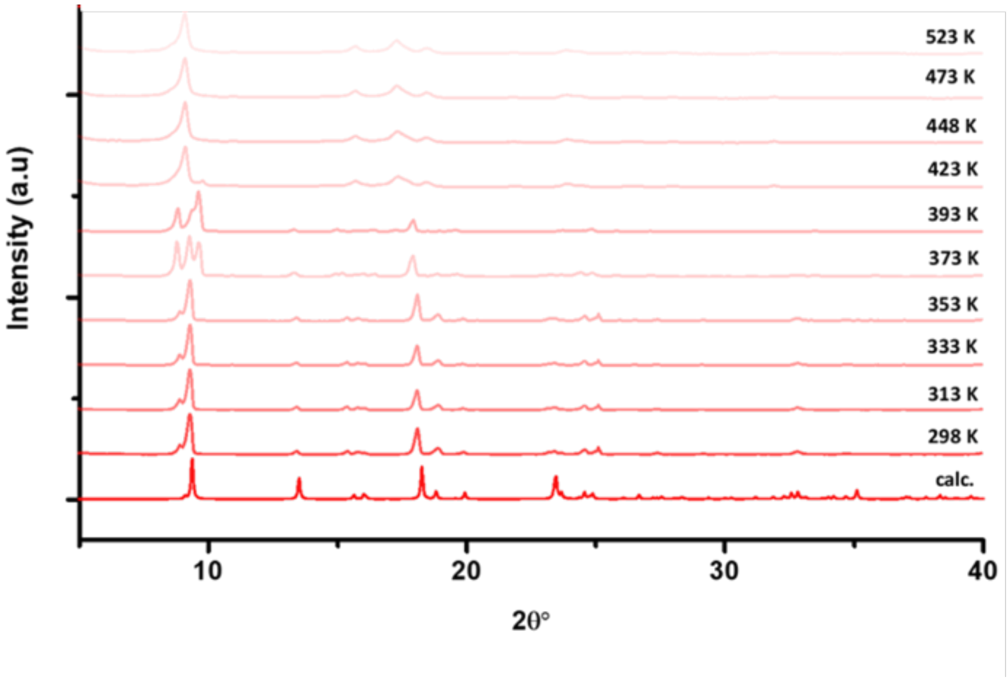

Figure S4: VT-PXRD diffractograms of **sql-Co-aqua** under N_2.

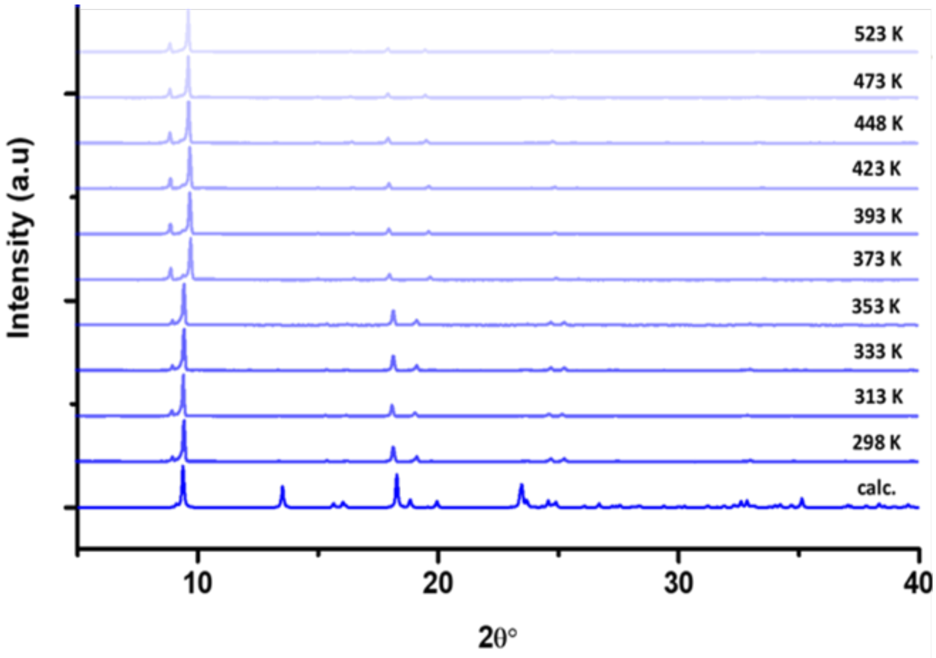

Figure S5: VT-PXRD diffractograms of **sql-Ni-aqua** under N_2.

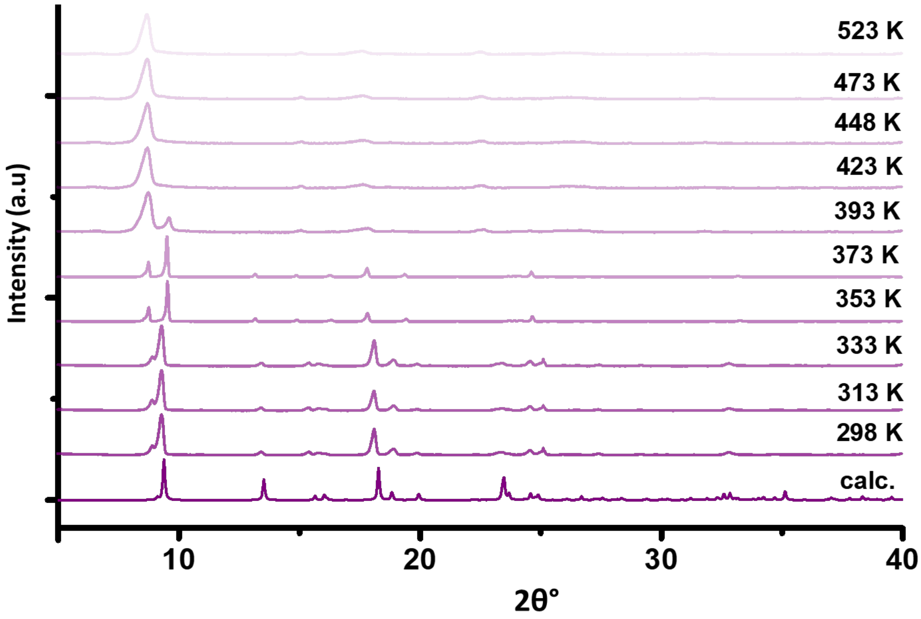

Figure S6: VT-PXRD diffractograms of **sql-Zn-aqua** under N_2.

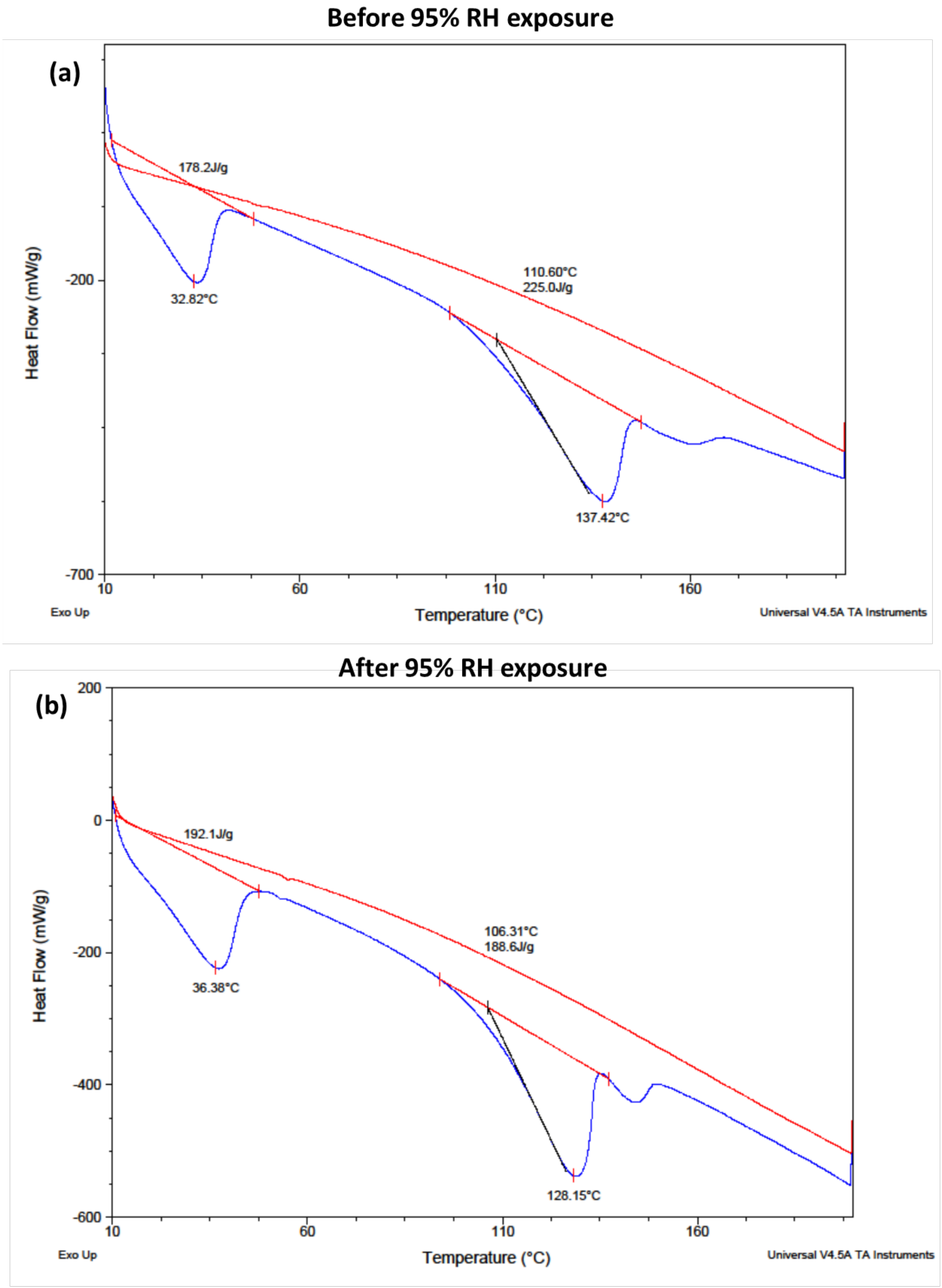

Figure S7: DSC thermograms of the heating segments for **sql-Zn-aqua** over two cycles (Cycle 1, blue line; Cycle 2, red line before (a) and after (b) exposure to 95% RH. The values were determined by integration of the two heating events regions (with respect to time)

Table S4: Selected crystallographic details for **sql-Zn-aqua-activated**

Compound	**sql-Zn-aqua-activated (CCDC# 2390774)**
Empirical formula	$C_{14}H_{12}N_2O_6Zn$
Formula weight	369.63
Temperature (K)	298 K (2)
Wavelength (Å)	0.71073
Crystal system	Monoclinic
Space group	*C*2/*c*
a(Å)	19.712(8)
b(Å)	11.673(5)
c(Å)	7.947(12)
β(°)	113.473(7)
V (Å^3)	1677.4(12)
Z	4
GOF on F^2	1.059
R_1, wR_2 [I>2s(I)]	0.0796, 0.1392
R_1, wR_2 (all data)	0.1673, 0.1622

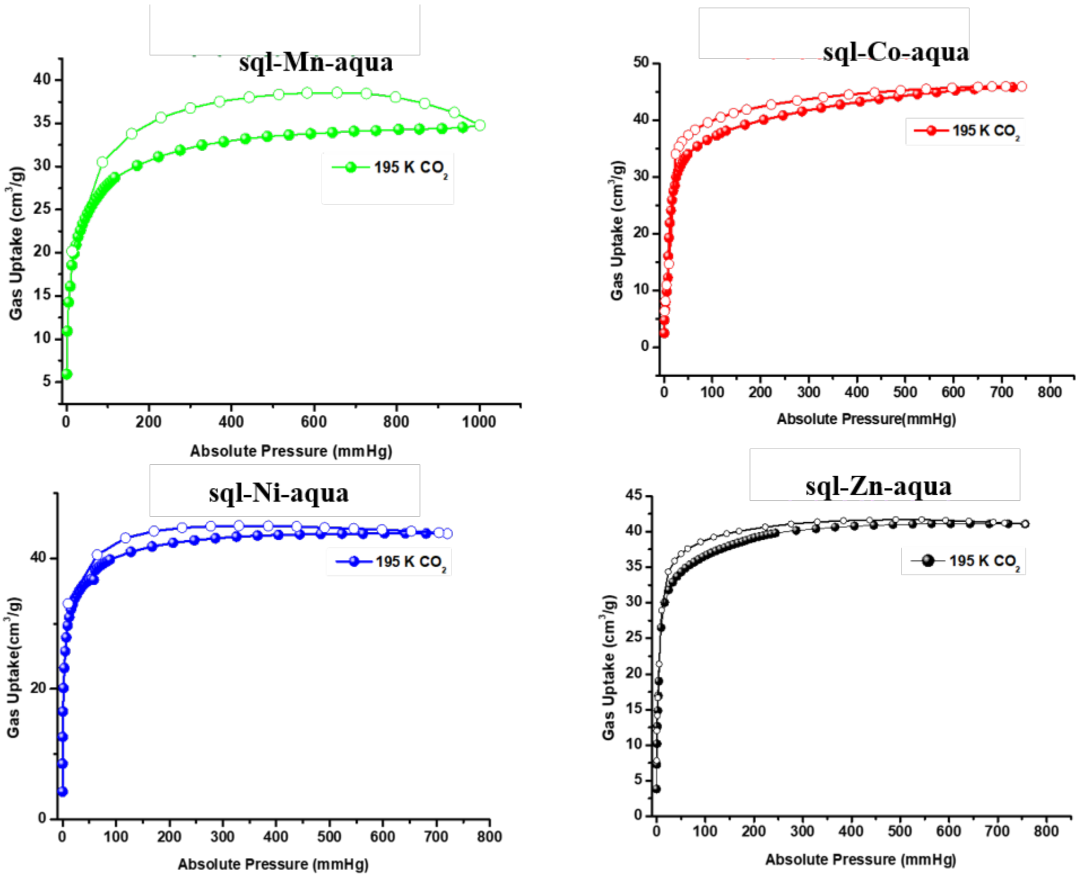

Figure S8: 195 K CO_2 adsorption and desorption isotherms measured for **sql-M-aqua** (M=Mn,Co,Ni,Zn).

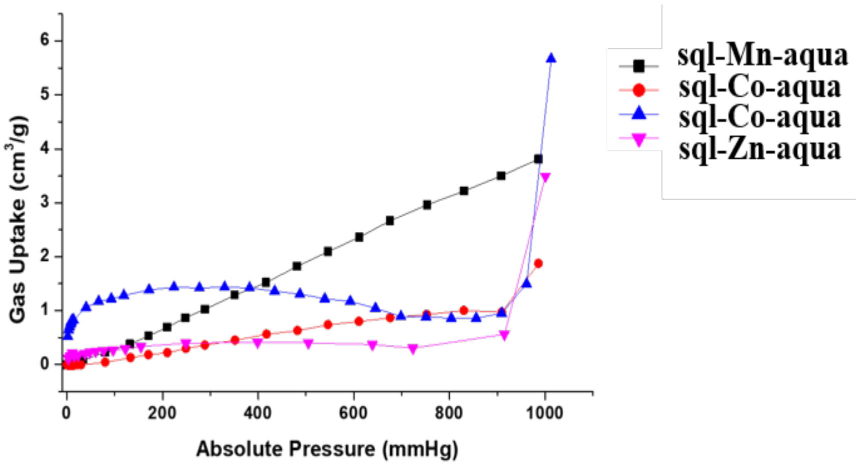

Figure S9: N_2 isotherms at 77K for **sql-M-aqua** (M=Mn,Co,Ni,Zn).

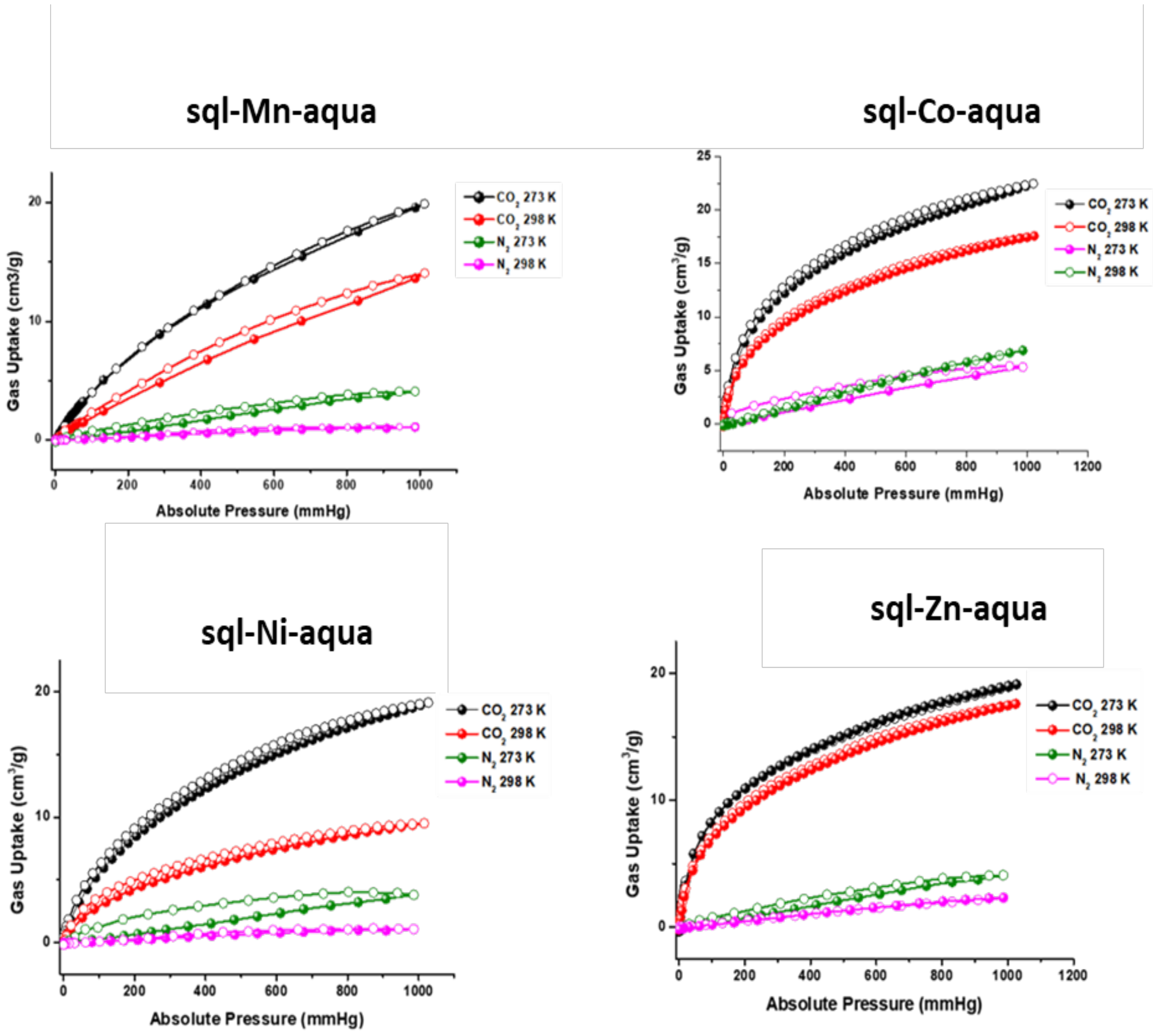

Figure S10. CO_2 and N_2 isotherms at 273 and 298 K for **sql-M-aqua** (M= Mn, Co, Ni, Zn).

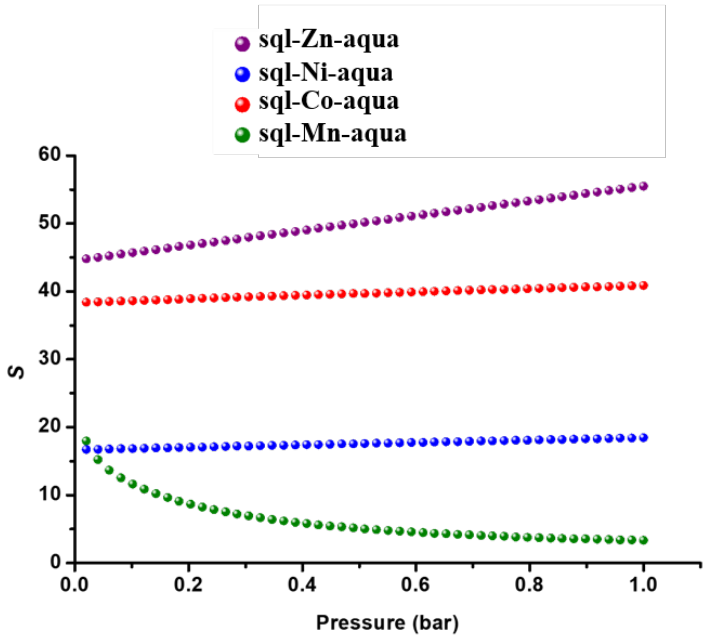

Figure S11. IAST selectivities of **sql-M-aqua** (M=Mn,Co,Ni,Zn) at 298 K for CO_2/N_2 and compositions of 15:85, plotted as a function of pressure.

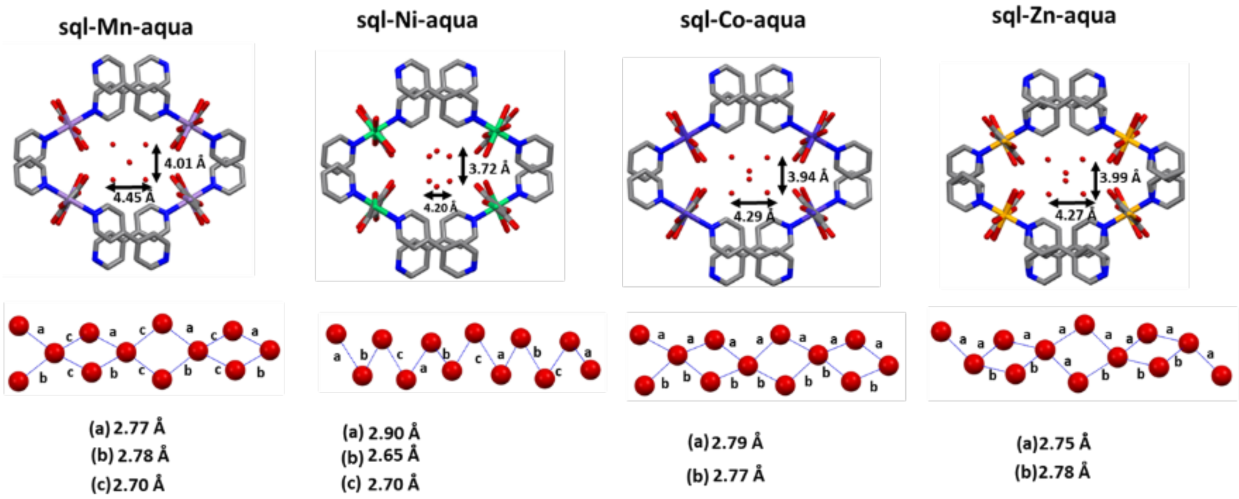

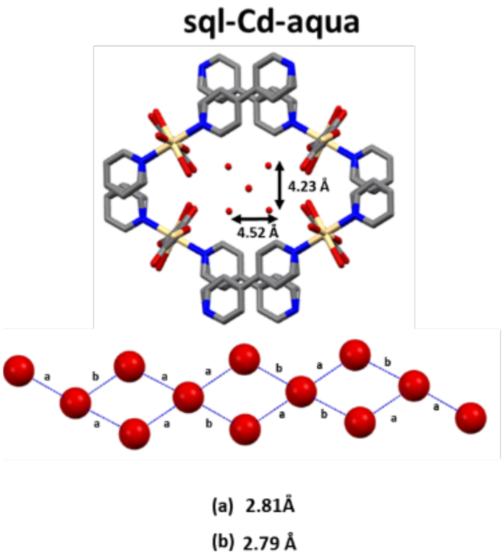

Figure S12: Comparison of guest-guest interactions in **sql-M-aqua** (Mn,Ni,Co,Zn,Cd).

Estimation of Adsorption Enthalpy using Clausius-Clapeyron Approach

The enthalpy of adsorption Δh_{ads} (per mole of host) with respect to H_2O loading (wt%) were determined using the Clausius-Clapeyron relation[14]

$$lnP = \Delta h_{ads}/R \cdot 1\ T + C$$

where the P is the partial pressure (Pa) of water at temperature T (K), R is the gas constant (8.314 kJ mol−1 K-1), and C is a constant. The values of Δh_{ads} were obtained from the isosteres of adsorption (Figure S12) derived from the first cycle of the three sorption isotherms at 15, 27 and 40 °C (Figures S9-S11).

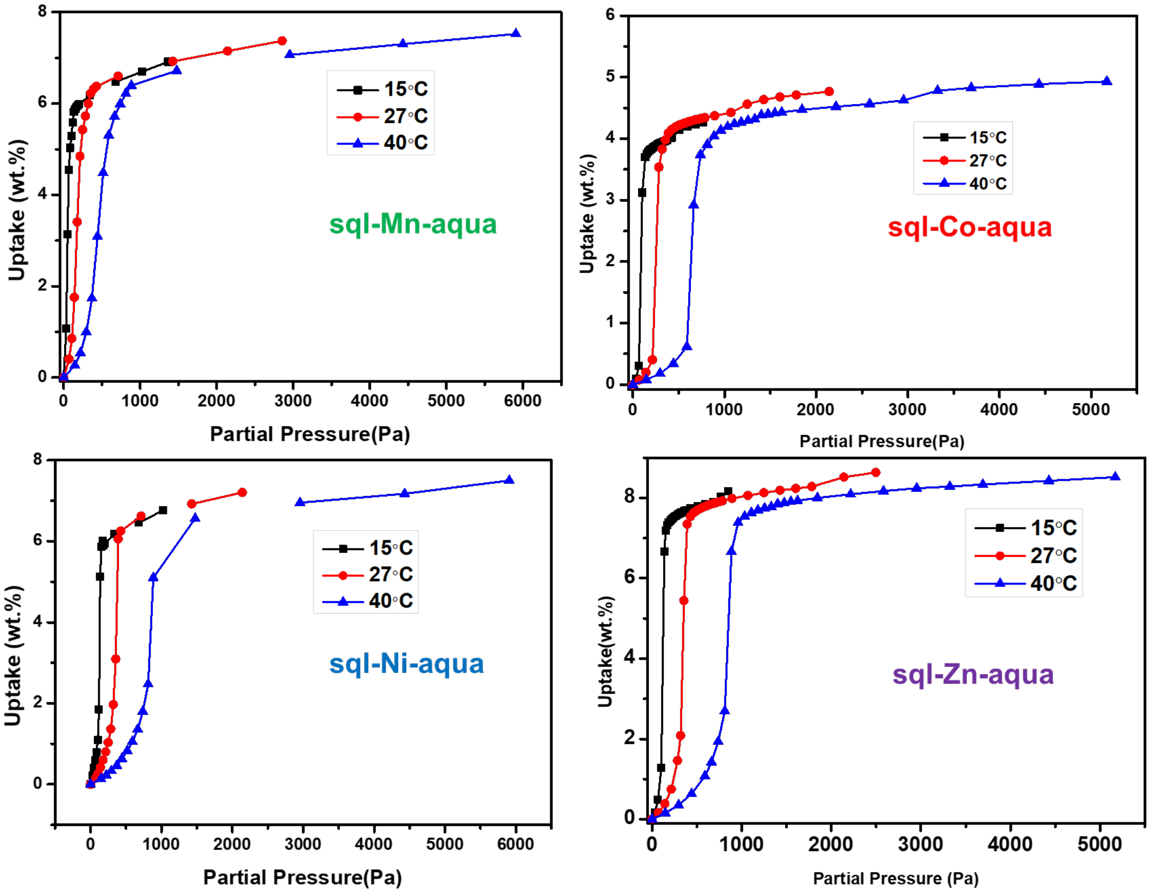

Figure S13: Water vapour sorption isotherms for (M= Mn, Co, Ni, Zn) measured at 10 °C, 27 °C and 40 °C.

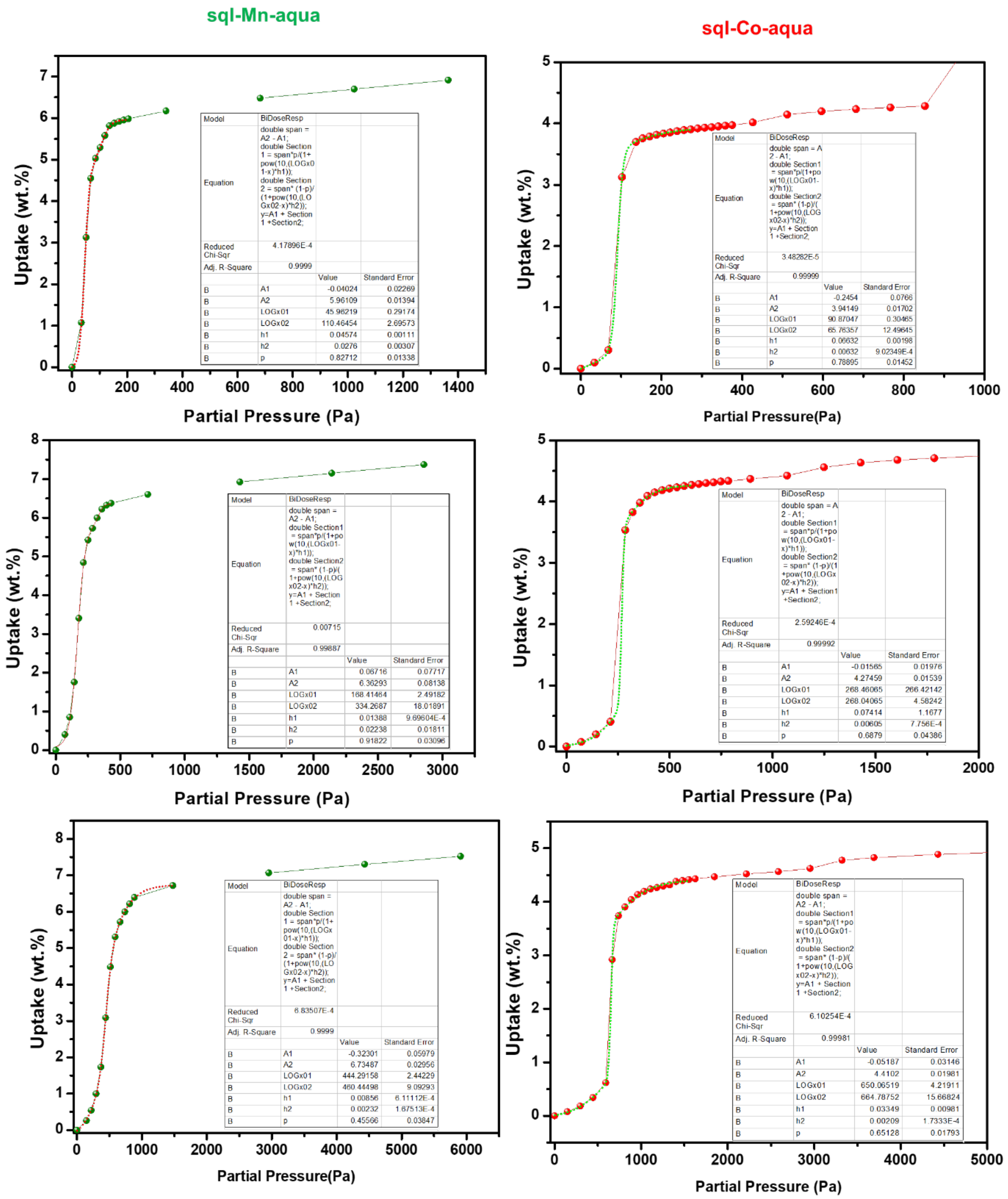

Figure S14: Water vapour adsorption isotherms used for calculating Δ*h* recorded at 10 °C (top), 27 °C (middle,) and 40 °C (bottom, red squares) using a dynamic vapour sorption analyser. Details regarding the fitting function to determine uptake as a function of partial pressure are shown as insets. The function is overlaid as red and green line for **sql-M-aqua** (M= Mn, Co) respectively.

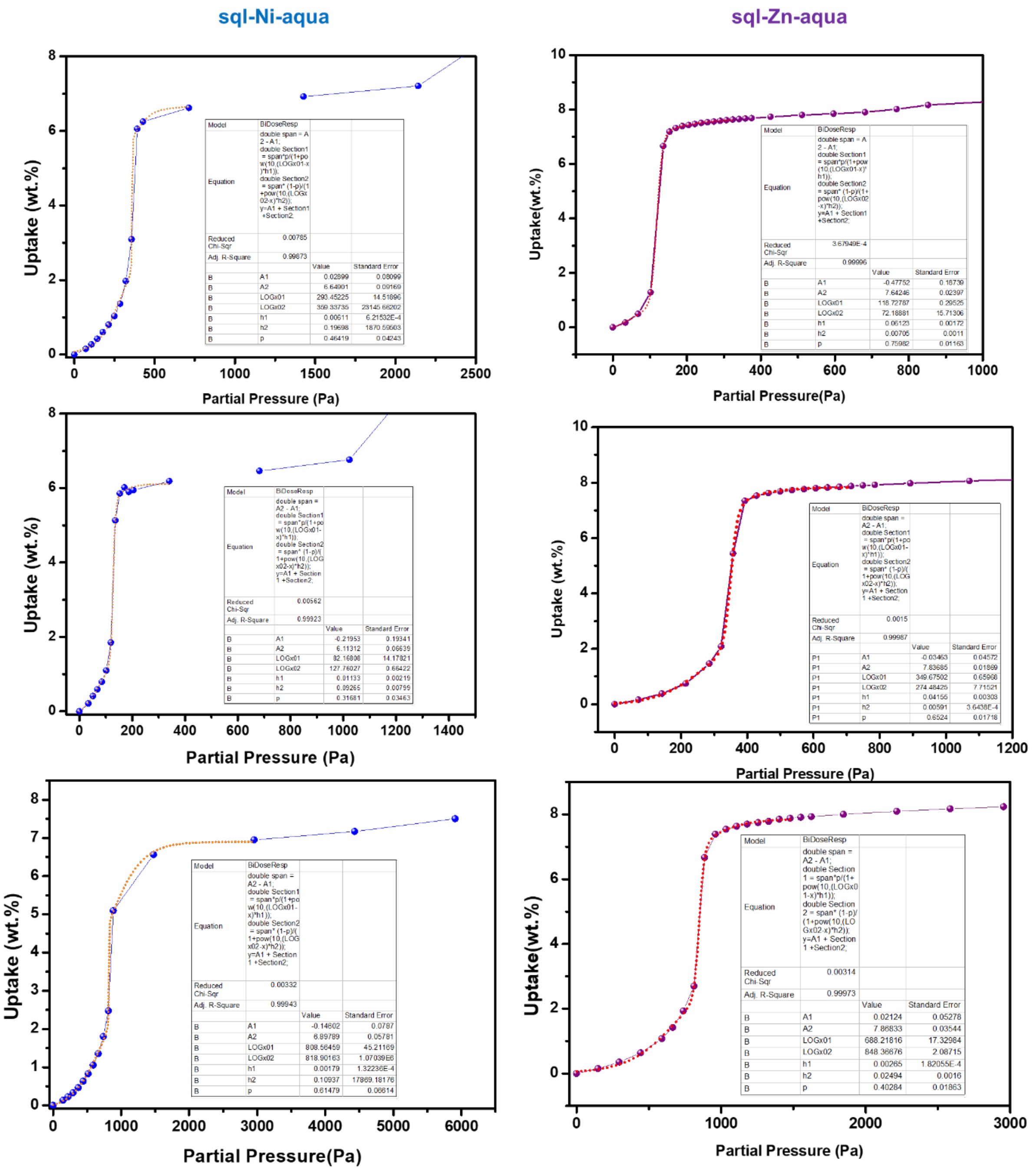

Figure S15: Water vapour adsorption isotherms used for calculating Δ*h* recorded at 10 °C (top), 27 °C (middle,) and 40 °C (bottom, red squares) using a dynamic vapour sorption analyser. Details regarding the fitting function to determine uptake as a function of partial pressure are shown as insets. The function is overlaid as a orange and red line for **sql-M-aqua** (M= Ni, Zn) respectively.

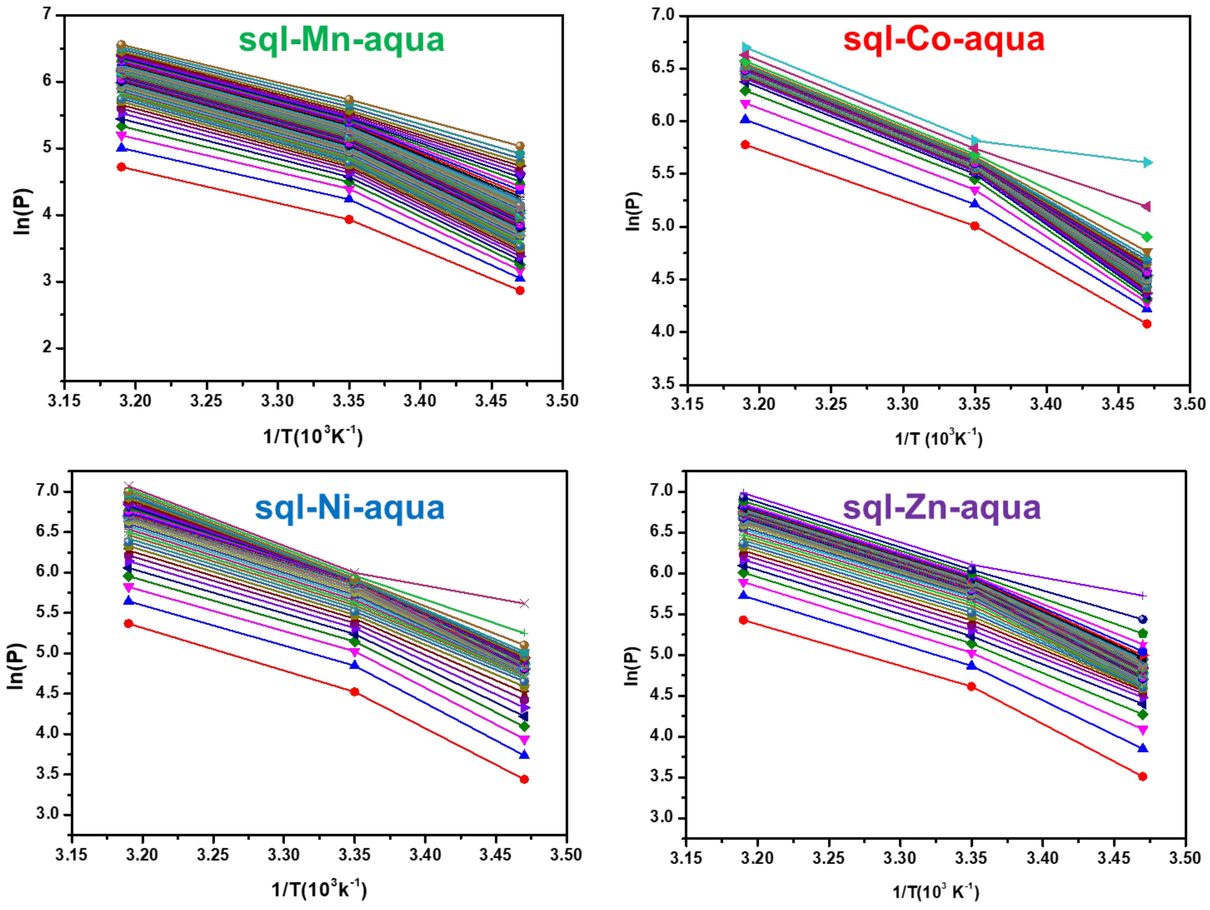

Figure S16: Adsorption isosteres used to calculate enthalpy of adsorption (Δ*h*) for **sql-M-aqua** (M= Mn, Co, Ni, Zn).

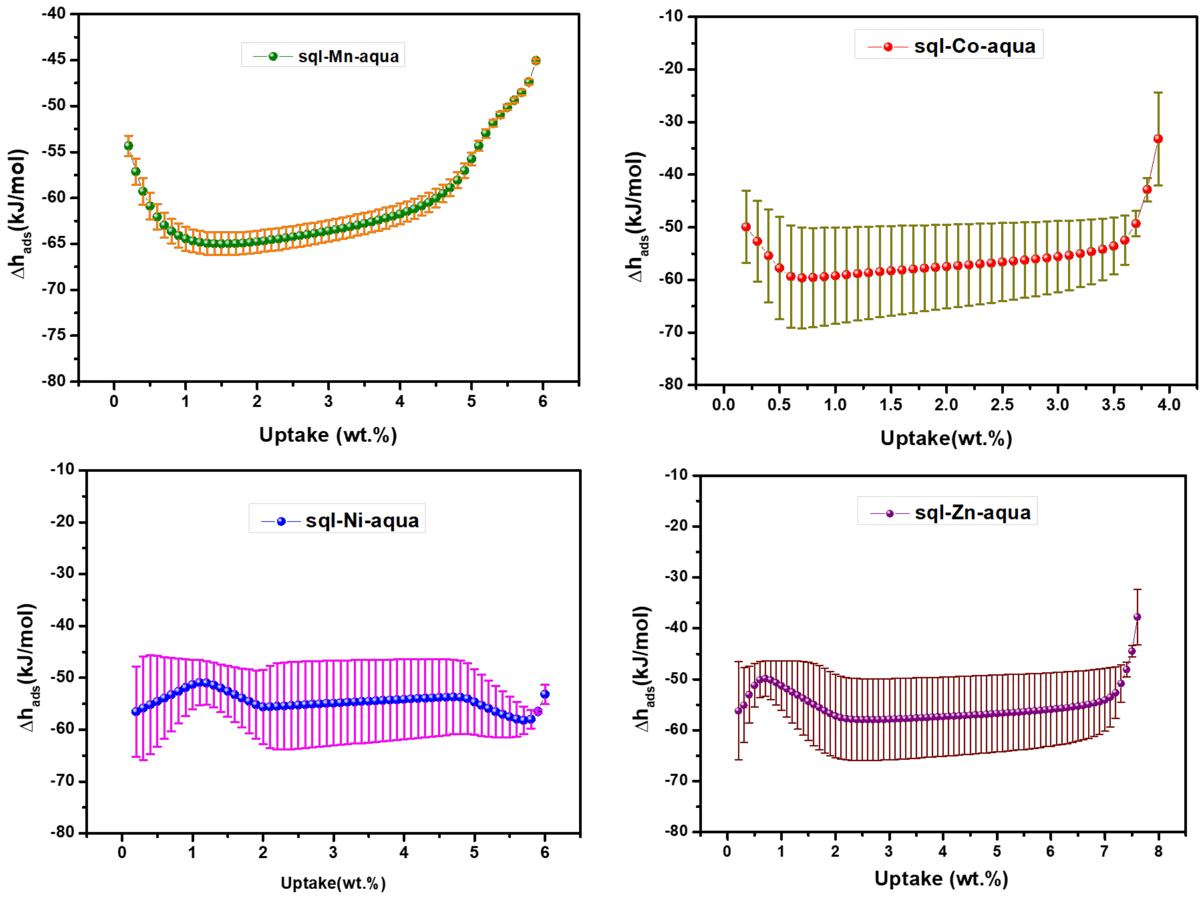

Figure S17: Enthalpy of adsorption (Δ*h*) with respect to water uptake for **sql-M-aqua** (M= Mn, Co, Ni, Zn).

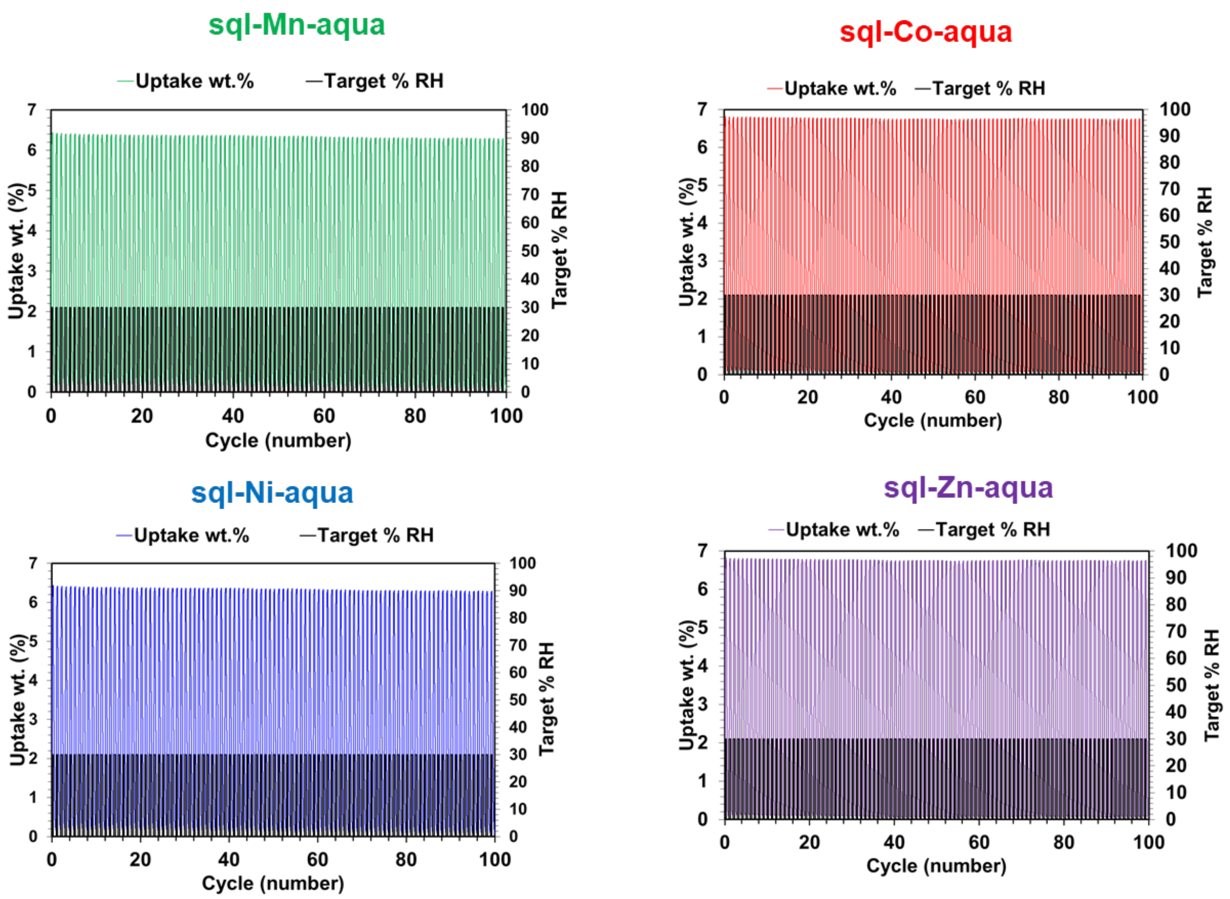

Figure S18: 100 regeneration cycles on a 11 mg sample for **sql-M-aqua** (M= Mn, Co, Ni, Zn).

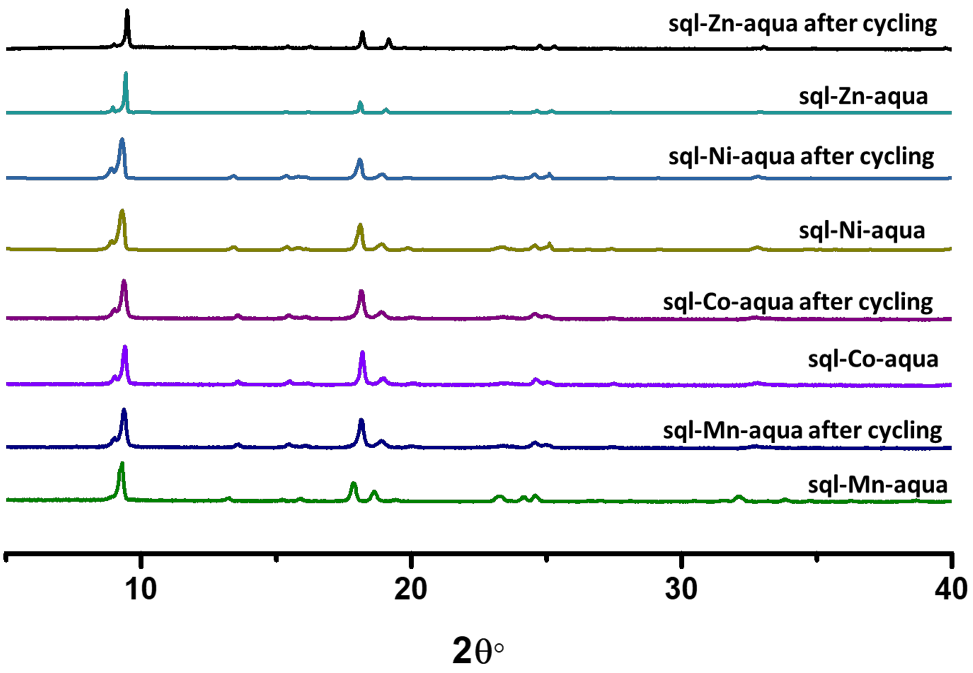

Figure S19: PXRD of **sql-M-aqua** (M=Mn,Co,Ni,Zn) after cycling experiment.

References

1. T. Francart, A. van Wieringen and J. Wouters, *Journal of Neuroscience Methods*, 2008, **172**, 283-293.
2. G. M. Sheldrick, *Acta Crystallographica Section C Structural Chemistry*, 2015, **71**, 3-8.
3. C. F. Macrae, I. Sovago, S. J. Cottrell, P. T. A. Galek, P. McCabe, E. Pidcock, M. Platings, G. P. Shields, J. S. Stevens, M. Towler and P. A. Wood, *Journal of Applied Crystallography*, 2020, **53**, 226-235.
4. O. V. Dolomanov, L. J. Bourhis, R. J. Gildea, J. A. K. Howard and H. Puschmann, *Journal of Applied Crystallography*, 2009, **42**, 339-341.
5. A. A. Bezrukov, D. J. O'Hearn, V. Gascon-Perez, S. Darwish, A. Kumar, S. Sanda, N. Kumar, K. Francis and M. J. Zaworotko, *Cell Reports Physical Science*, 2023, **4**.
6. I. J. Bruno, J. C. Cole, P. R. Edgington, M. Kessler, C. F. Macrae, P. McCabe, J. Pearson and R. Taylor, *Acta Crystallographica Section B*, 2002, **58**, 389-397.
7. C. Näther, J. Greve and I. Jeβ, *Chemistry of Materials*, 2002, **14**, 4536-4542.
8. J. Greve, I. Jeß and C. Näther, *Journal of Solid State Chemistry*, 2003, **175**, 328-340.
9. C.-C. Wang, S.-Y. Ke, K.-T. Chen, Y.-F. Hsieh, T.-H. Wang, G.-H. Lee and Y.-C. Chuang, *Crystals*, 2017, **7**, 364.
10. A. Kumar Ghosh, D. Ghoshal, E. Zangrando, J. Ribas and N. Ray Chaudhuri, *Dalton Transactions*, 2006, DOI: 10.1039/B510999J, 1554-1563.
11. S. C. Manna, E. Zangrando, J. Ribas and N. Ray Chaudhuri, *Inorganica Chimica Acta*, 2005, **358**, 4497-4504.
12. X.-J. Ke, D.-S. Li, J. Zhao, L. Bai, J.-J. Yang and Y.-P. Duan, *Inorganic Chemistry Communications*, 2012, **21**, 129-132.
13. S. Goswami, A. K. Mondal and S. Konar, *Journal of Chemical Sciences*, 2015, **127**, 257-264.
14. H. Kim, H. J. Cho, S. Narayanan, S. Yang, H. Furukawa, S. Schiffres, X. Li, Y. B. Zhang, J. Jiang, O. M. Yaghi and E. N. Wang, *Sci Rep*, 2016, **6**, 19097.
